# A Short Peptide Inhibitor as an Activity-Based Probe for Matriptase-2

**DOI:** 10.3390/ph11020049

**Published:** 2018-05-21

**Authors:** Martin Mangold, Michael Gütschow, Marit Stirnberg

**Affiliations:** Pharmaceutical Chemistry I, Pharmaceutical Institute, University of Bonn, Bonn 53113, Germany; martinmangold@uni-bonn.de (M.M.); marit.stirnberg@web.de (M.S.)

**Keywords:** matriptase-2, Activity-based probe, peptide inhibitor

## Abstract

Matriptase-2 is a type II transmembrane serine protease and a key regulator of systemic iron homeostasis. Since the activation mechanism and several features of the physiological role of matriptase-2 are not fully understood, there is strong need for analytical tools to perform tasks such as distinguishing active and inactive matriptase-2. For this purpose we present a short biotinylated peptide derivative with a chloromethyl ketone group, biotin-RQRR-CMK, as an activity-based probe for matriptase-2. Biotin-RQRR-CMK was kinetically characterized and exhibited a second-order rate constant of inactivation (*k*_inac_/*K*_i_) of 10,800 M^−1^ s^−1^ towards the matriptase-2 activity in the supernatant of transfected human embryonic kidney (HEK) cells. Biotin-RQRR-CMK was able to label active matriptase-2, as visualized in western blot experiments. Pretreatment with aprotinin, an active-site directed inhibitor of serine proteases, protected matriptase-2 from the reaction with biotin-RQRR-CMK.

## 1. Introduction

Proteases play an important role in the catalysis and regulation of numerous physiological events in the human body. These enzymes can be divided into different classes based on the nature of their catalytic mechanisms. Serine proteases constitute the largest class whose enzymatic reactions are mediated by the serine residue in their active site. This group includes enzymes such as thrombin and trypsin, which are critical in the process of blood coagulation and digestion. Our enzyme of interest, matriptase-2 (MT-2), a member of the type II transmembrane serine proteases (TTSPs) [1,2], is mainly expressed in the liver [3] and considered as a key regulator of iron homeostasis [4,5]. By cleaving membrane-bound hemojuvelin (HJV), the co-receptor of the bone morphogenic protein (BMP) receptor, MT-2 decreases the activation of the SMAD signaling cascade and, in consequence, the expression of the hepcidin gene *Hamp* [6,7]. Hepcidin regulates intestinal iron absorption by binding to the iron importer ferroportin, leading to its internalization and degradation [8]. Therefore, high expression levels of MT-2 result in increased iron uptake, which led to the proposition of MT-2 as a target for the treatment of iron overload caused by low hepcidin levels [9,10,11,12]. In contrast, several mutations in the MT-2 gene *TMPRSS6* have been reported to cause an inherited form of anemia in humans, referred to as iron refractory iron deficieny anemia (IRIDA) [13]. This disease is characterized by reduced plasma iron levels which cannot be influenced by the oral treatment with iron. 

MT-2 is synthesized as an 802 amino acid zymogen with a molecular weight of 88.9 kDa [1]. Similar to other TTSPs, MT-2 is comprised of a short N-terminal cytoplasmic tail, a transmembrane domain, a stem region consisting of two CUB and three LDLRA domains and the C-terminal trypsin-like serine protease domain. This catalytic domain possesses a high similarity to that of the closely related enzyme matriptase (45%). It contains the catalytic triad ^57^His, ^102^Asp and ^195^Ser (chymotrypsinogen numbering) as well as the activation sequence Arg-Ile-Val-Gly-Gly which plays an important role in zymogen activation. The autocatalytic cleavage of the Arg-Ile bond results in the active two-chain form of MT-2 which is still connected by a disulfide bridge between two cysteine residues in the LDLRA and catalytic domains, respectively. A second cleavage in the CUB domain leads to the release of an enzymatically active, approximately 55-kDa protein fragment into the supernatant. This fragment consists of the catalytic and the three LDLRA domains of MT-2 [14]. 

**Figure 1:**
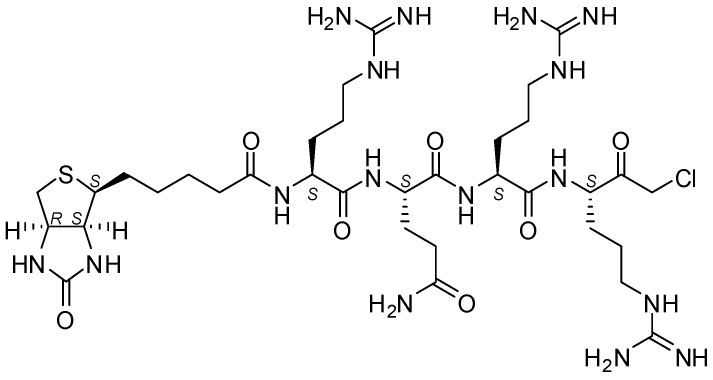
Structure of biotin-RQRR-CMK.

Since the underlying mechanism of these processing steps is not yet fully understood, there is strong need for analytical tools that are capable of distinguishing active and inactive MT-2. As a biochemical tool compound, an activity-based probe could thus be of vital importance for the MT-2-related research. This is even more the case as specific antibodies for MT-2 are lacking. Since MT-2 prefers substrates with arginine at P1, P2 or P3 and P4 positions [9,15,16,17], this knowledge can and has been used to design small peptide inhibitors and peptidomimetic activity-based probes. Already described low-molecular weight MT-2 inhibitors include, for example, amidinophenylalanine derivatives [18], peptidic ketones [19,20], sunflower trypsin inhibitor-1 (SFTI-1) analogues [21], and bisbenzamidines [22,23,24], the latter type of compounds also being established as fungicides and antiprotozoal agents [25,26,27] and able to bind to the double strand DNA [28,29]. Here we present a short biotinylated peptide probe with a chloromethyl ketone warhead (biotin-RQRR-CMK) as an irreversible inhibitor and activity-based probe for MT-2 (Figure 1). This probe was originally developed for the related enzyme matriptase [30,31], which exhibits a similar substrate specificity [32]. Selectivity for MT-2 over matriptase has rarely been observed within series of synthetic inhibitors [9,18,23,24], but has been achieved with certain SFTI-1 analogues [21] and, in particular, peptidic ketones containing unnatural amino acids, such as l-*allo*-threonine at P2 position [20]. Its side chain may favorably interact with ^99^His of the S2 pocket of MT-2, while matriptase has Phe at this position [19,20,33]. 

Biotin-RQRR-CMK was obtained from American Peptide Company. Such arginine-containing chloromethyl ketones are synthetically accessible by converting orthogonally protected arginine. N_α_-Boc-arginine, via mixed anhydride activation, can be reacted with diazomethane and subsequently with hydrogen chloride dissolved in ethanol or ether. The resulting N_α_-deprotected chloromethyl ketone derivative can be coupled to a peptide portion. The final deprotection of the nitro- and 2,3,6-trimethyl-4-methoxybenzenesulfonyl-protected guanidine group(s) can be accomplished with anhydrous hydrogen fluoride [34] and trifluoroacetic acid [35,36], respectively, in the presence of (thio)anisole. 

## 2. Results

### 2.1. Kinetic Evaluation of Biotin-RQRR-CMK As an Inhibitor of MT-2

We used stably transfected human embryonic kidney (HEK) cells as the source for MT-2 [14]. Wild-type HEK cells do not produce MT-2. Initially, the inhibitory potency of biotin-RQRR-CMK against MT-2 was assessed. For this purpose, we determined MT-2 activity in the supernatant of transfected HEK cells using a tripeptidic fluorogenic substrate. Probe concentrations of 25 nM to 100 nM were applied. Values *k*_obs_ were obtained from non-linear regression of the progress curves of the supernatant experiment using the equation

[P] = v_0_ (1 − e^−*k*obs t^)/*k*_obs_ + d (1)

where [P] is the product concentration, v_0_ is the initial rate and d is the offset. Values *k*_inac_/*K*_i_ were obtained using the equation

**Figure 2:**
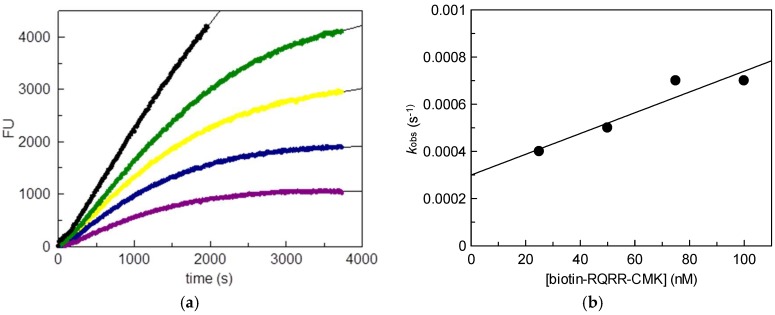
Matriptase-2 (MT-2) activity in the supernatant of transfected human embryonic kidney (HEK) cells in the presence of increasing concentrations of biotin-RQRR-CMK: (**a**) Fluorescence units (FU) plotted vs. time. Colored progress curves represent reactions in the presence of different probe concentrations (● uninhibited reaction; 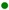 25 nM; 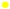 50 nM; 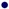 75 nM; 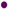 100 nM); (**b**) Values *k*_obs_ plotted vs. probe concentrations.

*k*_inac_/*K*_i_ = (*k*_obs_/[biotin-RQRR-CMK]) (1 + ([S]/*K*_m_)) (2)

where *k*_inac_ is the first-order inactivation rate constant, *K*_i_ is the inhibition constant, [biotin-RQRR-CMK] is the probe concentration, [S] is the substrate concentration and *K*_m_ is the Michaelis constant. A concentration dependent decrease in MT-2 activity was observed in the course of the experiment (Figure 2). The progress curves reflected an irreversible binding behavior of the probe exhibiting a *k*_inac_/*K*_i_ value of 10,800 M^−1^ s^−1^.

**Figure 3:**
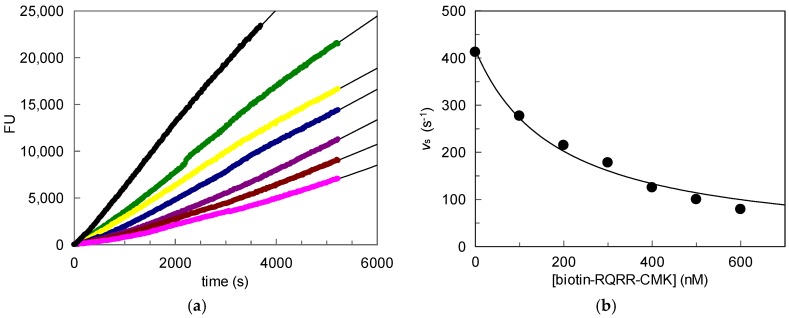
MT-2 activity on the surface of transfected HEK cells in the presence of increasing concentrations of biotin-RQRR-CMK: (**a**) Fluorescence units (FU) plotted vs. time. Colored progress curves represent reactions in the presence of different probe concentrations (● uninhibited reaction; 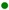 100 nM; 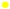 200 nM; 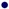 300 nM; 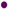 400 nM; 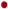 500 nM; 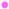 600 nM); (**b**) Values v_s_ plotted vs. probe concentrations.

To further investigate the inhibitory potency of the probe in an in vitro approach, MT-2 activity was measured on the surface of stably transfected HEK cells expressing MT-2. Different probe concentrations were applied (Figure 3). It is worth noting that the progress curves displayed a linear shape over the whole course of the experiment, indicating that a time-dependent loss of enzymatic activity did not occur, in contrast to the supernatant experiments. Still, a concentration-dependent decrease of MT-2 activity was observed, but overall higher probe concentrations were needed to achieve inhibition. A *K*_i_ value of 89 nM was obtained from linear regression of the progress curves and by using the equation

*K*_i_ = [I]/(((v_0_/v_i_) − 1)(1 + ([S]/*K*_m_))) (3)

where v_i_ is the rate in the presence of the inhibitor. 

### 2.2. Detection of Active MT-2 in Western Blot Experiments

**Figure 4:**
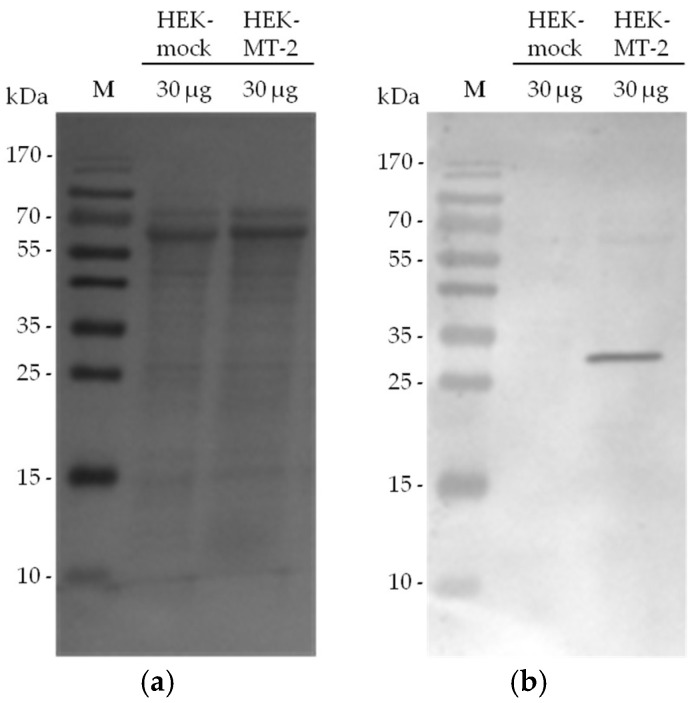
MT-2 detection using an antibody against the myc tag of the transfected protein: (**a**) Ponceau S staining after protein transfer to a nitrocellulose membrane; (**b**) Detection of MT-2 by an anti-myc antibody.

In our experimental set up, MT-2 can be visualized in western blot experiments in the culture supernatant of HEK cells by using an antibody against the myc tag of the transfected protein. We investigated 30-µg samples from supernatant of HEK cells expressing MT-2 or an empty vector (mock) to assess the presence of MT-2. A Ponceau S staining of the blot is included as reference for total protein amounts (Figure 4: panel a). MT-2 was detected as an approximately 30-kDa protein after running the SDS gel under reducing conditions and applying the anti-myc antibody (Figure 4: panel b). This band represents the catalytic domain of the enzyme. The corresponding band was lacking in the culture supernatant of HEK-mock cells expressing an empty vector. 

**Figure 5:**
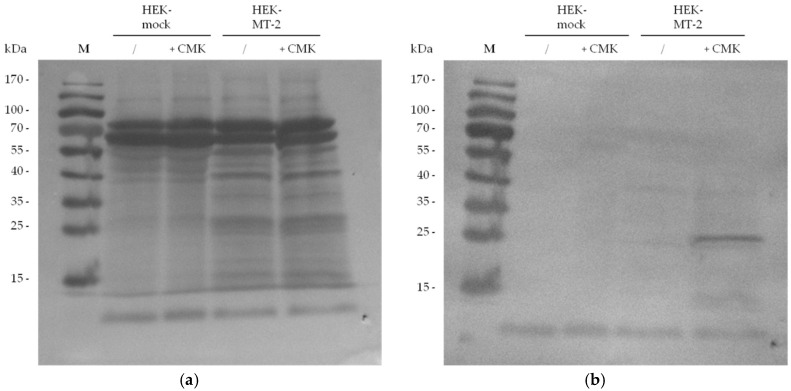
MT-2 detection after incubation with biotin-RQRR-CMK (50 µM, 1 h): (**a**) Ponceau S staining after protein transfer to a nitrocellulose membrane; (**b**) Detection of MT-2 by biotin-RQRR-CMK after incubation with Strep-Tactin/AP conjugate.

Similar to antibody staining addressing the myc tag, the biotin tag which was chemically introduced by biotin-RQRR-CMK was employed for the detection of MT-2 after western blotting. For this purpose, 30 µg of HEK-mock or HEK-MT-2 supernatants were incubated with or without biotin-RQRR-CMK prior to SDS-PAGE. After transfer to a nitrocellulose membrane, the blot was incubated with a conjugate of Strep-Tactin with alkaline phosphatase (AP). MT-2 was visualized as an approximately 30-kDa protein band in accordance to the anti-myc staining (Figure 5). This band was only observed in the supernatant of HEK cells expressing active MT-2 that was treated with the probe.

**Figure 6:**
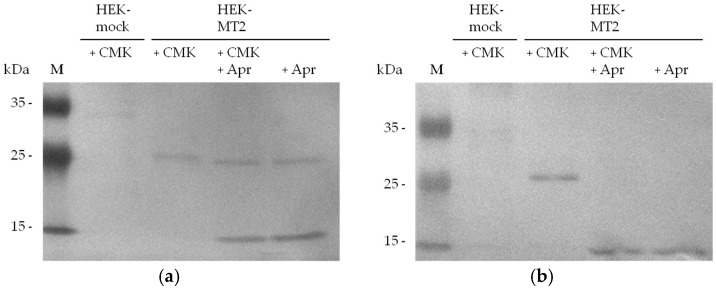
MT-2 detection by biotin-RQRR-CMK after treatment of 30 µg HEK cell supernatant with 50 µM aprotinin for 2 h before probe treatment (5 µM, 30 min): (**a**) Detection of MT-2 by an anti-myc antibody; (**b**) Detection of MT-2 by biotin-RQRR-CMK after incubation with Strep-Tactin/AP conjugate.

To confirm that biotin-RQRR-CMK binds to the active site of MT-2, we added aprotinin to the supernatants of HEK-MT-2 cells. Treatment with aprotinin or DMSO was carried out 2 h prior to probe treatment. Aprotinin, a known inhibitor of serine proteases [37], has been shown to inhibit MT-2 [1,38]. MT-2 was present in the supernatant of transfected HEK cells incubated with the probe and/or aprotinin (Figure 6: panel a). Detection of the protease-probe complex after SDS-PAGE and western blotting showed the labeled MT-2 fragment which was missing in HEK-MT-2 supernatants after aprotinin treatment (Figure 6: panel b).

## 3. Discussion and Conclusions

MT-2 is a type II transmembrane serine protease that is shed into the supernatant after two autocatalytic activation steps [14]. The shed form of the enzyme is still enzymatically active and can be used to measure MT-2 activity in the supernatant of transfected cells. Our findings reveal a short peptide probe as an irreversible inhibitor and activity-based probe for MT-2. This compound was first developed as an activity-based probe of the closely related enzyme matriptase and consists of a biotin tag, a peptide linker (Arg-Gln-Arg-Arg) and a chloromethyl ketone warhead [30]. Due to its three arginine residues, we predict that biotin-RQRR-CMK occupies the P1, P2 and P3/P4 binding pockets of MT-2 [16,17], while the chloromethyl ketone group forms an irreversible bond with the active-site serine, thereby inhibiting the enzyme. This is reflected by the results of the kinetic experiments we performed. The probe was able to irreversibly inhibit MT-2 in the supernatant of transfected HEK cells in a concentration-dependent manner in the nanomolar range. The irreversible binding behavior could only be observed by measuring MT-2 activity in cell culture supernatants, but not by following the surface-associated proteolytic activity. This finding reflects the opportunity that the intact HEK cells can still produce and shed new MT-2, as well as possibly internalize the inhibited enzyme, thereby generating a continuous flow of enzymatic activity. Nonetheless, the cell surface measurements also revealed a concentration-dependent inhibition of MT-2 activity and a two-digit nanomolar *K*_i_ value of the probe. 

In further experiments we showed that the biotin tag of the activity-based probe can be used to detect MT-2 in the supernatant of transfected HEK cells. We were able to selectively label and detect MT-2 in the form of a single approximately 30-kDa protein band in a complex protein mixture. The absence of this protein band in HEK-mock supernatant, as well as a protein band of similar mobility after myc-tag detection both indicate that it represents the catalytic domain of MT-2. This also supports our prediction of an irreversible bond between MT-2 and the tested probe that withstands the reducing conditions of the SDS loading buffer and the process of SDS-PAGE and western blotting. Inhibition of MT-2 with the serine protease inhibitor aprotinin prior to probe treatment protected the enzyme from the reaction with biotin-RQRR-CMK and thus prevented the detection of the MT-2 fragment, confirming that inhibition is achieved by binding to the enzyme’s active site.

With a high *k*_inac_/*K*_i_ value of 10,800 M^−1^ s^−1^ the probe exhibits a high affinity for MT-2 and can be used to detect the active enzyme. Since endogenous MT-2 is expressed at very low levels in hepatocyte cell cultures (data with HepG2 cells are not shown) and its active form constitutes only a part of total MT-2 protein content, an overexpression system was applied to supply a sufficient amount of protein. This is a common problem in MT-2-related research. Further structural modifications of the probe presented might pave the way to a tool compound powerful enough to label even the small amounts of active MT-2 in endogenous systems. Such a probe might also circumvent the lack of specific antibodies against MT-2. Even though biotin-RQRR-CMK is not selective for MT-2 over matriptase, it can be considered a viable tool for the screening and visualization of active MT-2, since the expression patterns of both enzymes are different [3,39].

## 4. Materials and Methods

### 4.1. Preparation of HEK-Cell Supernatant

Stably transfected human embryonic kidney (HEK) cells expressing MT-2 or an empty vector construct were cultivated in Dulbecco’s Modified Eagle’s Medium (DMEM) containing fetal bovine serum (FBS) as well as penicillin and streptadivin in a concentration of 100 mg/mL at 37 °C. After a confluence of approximately 70% was reached the culture medium was discarded and the cells were washed two times with phosphate buffered saline (PBS). After a further incubation step for two days at 37 °C in improved Minimal Essential Medium (Opti-MEM) the culture supernatant was collected and centrifuged at 2000 g and 4 °C for 10 min to separate cells. The supernatant was then concentrated by the use of 10-kDa size exclusion Amicon Ultra centrifugal filters at 4 °C and 7500 g. The protein concentration of the obtained protein mixture was determined by absorption measurements using Roti-Nanoquant Bradford solution.

### 4.2. Kinetic Measurement of MT-2 Activity

Kinetic measurements were performed on a Fluostar Optima plate reader (BMG Lab Tech, Offenburg, Germany) in 96 well plates. MT-2 assay buffer (NaCl 150 mM, TRIS 50 mM, pH 8.0), a Boc-Gln-Ala-Arg-AMC substrate in a final concentration of 40 µM and different amounts of biotin-RQRR-CMK in DMSO were added into subsequent wells. The concentration of DMSO in each well was adjusted to the same level before the reaction was started by adding 10 µg of MT-2 supernatant to each well. The conversion of the substrate by MT-2 was followed over a time of 50 min at 37 °C with an excitation wavelength of 340 nm and an emission wavelength of 460 nm. 

### 4.3. Cultivation and Kinetic Measurement of MT-2 Expressing HEK Cells

To measure MT-2 activity at the cell surface, HEK cells were seeded in 96 well cell culture plates and cultivated at 37 °C in DMEM medium until a confluence of approximately 70% was reached. After two washing steps with PBS and two further days of incubation in Opti-MEM medium at 37 °C, the culture medium was removed and the cells were washed two times with PBS. MT-2 assay buffer, biotin-RQRR-CMK and DMSO were then directly added to the cells before the reaction was started by adding substrate solution in a final concentration of 40 µM to each well. The conversion of the substrate by MT-2 was followed over a time of 50 min at 37 °C. 

### 4.4. Western Blot Detection of Myc-Tagged MT-2

HEK-cell supernatant was mixed with reducing sample buffer and incubated for 5 min at 95 °C. The protein mixture was separated by SDS-PAGE and blotted to a nitrocellulose membrane for 1 h at 67 V. The membrane was incubated in a blocking solution of 5% milk powder (MP) in Tris buffered saline containing 0.1% Tween solution (TBST) for 1 h and washed two times with TBST, followed by an incubation step in 5% MP/TBST with murine anti-myc antibody (dilution of 1:200) overnight at 4 °C. Afterwards the blot was washed two times with TBST, followed by an incubation step in TBST with secondary goat anti-mouse IgG antibody conjugated to AP (dilution 1:5000) for 1 h at room temperature. The blot was washed again, one time with TBST and two times with Tris buffered saline. The antibody was visualized the by incubation in 20 mL reaction buffer (MgCl_2_ 5 mM, NaCl 100 mM, TRIS 100 mM, pH 8.8) containing 7.5% 4-nitro blue tetrazolium chloride and 20% 5-bromo-4-chloro-3-indolyl phosphate.

### 4.5. Biotin-RQRR-CMK

The probe was obtained from America Peptide Company (San Diego, CA, USA). As notified by the provider, the peptide content was 65.9% and the peptide purity (HPLC) was 95.9%. HPLC conditions were as follows, instrument Waters 2695 (Milford, MA, USA), C18 column, wavelength 215 nm, 45 °C, flow rate 1.0 mL/min, buffer A: 0.1% TFA in H_2_O, buffer B: 0.1% TFA in MeCN, gradient (linear): 5–45% buffer B in 20 min, retention time: 8.35 min. LC/MS: m/z = 874.77, 872.77 ([M]^+^), 837.30 ([M − Cl]^+^).

### 4.6. Western Blot Detection of Biotin-RQRR-CMK-Labeled MT-2

HEK-cell supernatant was incubated with biotin-RQRR-CMK in a final concentration of 5 or 50 µM for 0.5 or 1 h at 37 °C. Pretreatment with aprotinin (Roth, Karlsruhe, Germany) in a final concentration of 50 µM was performed 2 h before application of the probe. After the addition of the reducing sample buffer, the protein mixture was separated by SDS-PAGE and blotted to a nitrocellulose membrane for 1 h at 67 V. Protein transfer was confirmed by Ponceau S staining. The membrane was incubated in a blocking solution of 3% bovine serum albumin in PBS containing 0.1% Tween solution (PBST) for 1 h and washed two times with PBST, followed by an incubation step in PBST with Strep-Tactin/AP conjugate (dilution of 1:5000) overnight at 4 °C. The blot was washed again, one time with PBST and two times with PBS. To visualize the Strep-Tactin/AP-probe complex, the membrane was incubated in 20 mL reaction buffer containing 7.5% 4-nitro blue tetrazolium chloride and 20% 5-bromo-4-chloro-3-indolyl phosphate.
